# Print–Pause–Print Fabrication of Tailored Electrochemical Microfluidic Devices

**DOI:** 10.1021/acs.analchem.3c03364

**Published:** 2023-12-14

**Authors:** Juan F. Hernández-Rodríguez, Daniel Rojas, Alberto Escarpa

**Affiliations:** † Department of Analytical Chemistry, Physical Chemistry and Chemical Engineering, University of Alcalá, Alcalá de Henares, 28805 Madrid, Spain; § Chemical Research Institute “Andres M. Del Rio”, University of Alcalá, Alcalá de Henares, 28805 Madrid, Spain

## Abstract

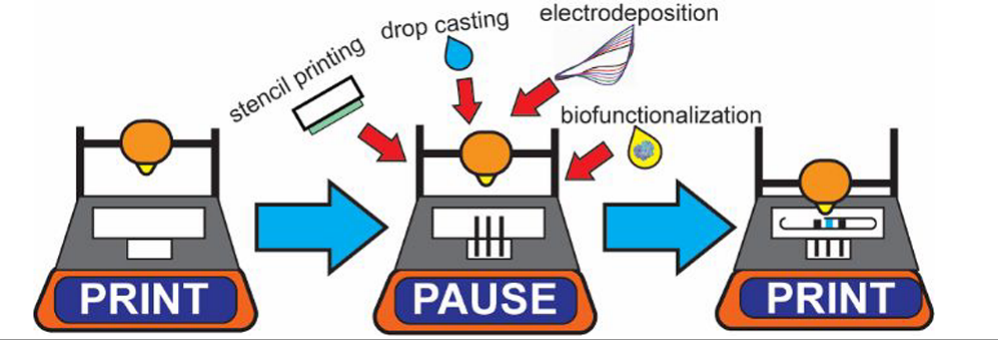

Three-dimensional (3D) printing technology has emerged as a powerful technology for the fabrication of low-cost microfluidics. Nevertheless, the fabrication of microfluidic devices integrating high-performance electrochemical sensors in practical applications is still an open challenge. Although automatic fabrication of the microfluidic device and the electrodes can be successfully carried out using a one-step multimaterial fused filament fabrication (FFF) approach, the as-printed electrochemical performance of these electrodes is not good enough for chemical (bio)sensing and their surface modification is challenging because after closing the channel there is no physical access to the electrode. Thus, here a pause–print–pause (PPP) microfabrication approach was implemented. The fabrication was paused before printing the microfluidics, and the filament-based electrodes were directly modified on the printing bed via stencil printing, drop casting, and electrodeposition. To exemplify this versatile workflow, the design of a microfluidic glucose sensor was proposed. To this end, first, the working and counter electrodes were stencil printed with graphite ink while the reference electrode was stencil printed with Ag|AgCl ink. Then, Prussian blue was formed on the working electrode either by drop casting or by electrodeposition, and glucose oxidase was drop cast on top. At this point, the microfabrication process was resumed, and the microfluidics were printed on top of the modified electrodes to complete the construction of hybrid electrochemical fluidic fused filament fabricated devices (h-eF^4^Ds). This print–pause–print approach is not limited to ink-based electrodes or glucose oxidase, and we envisage these results will pave the way for the effective integration of electrodes in microfluidic devices in a simple and clean-room-free approach, allowing the development of highly customized eF^4^Ds for a plethora of analytes with high significance.

## 1 Introduction

Additive manufacturing is a set of fabrication technologies that are revolutionizing the traditional microfluidics fabrication workflow and bringing unprecedented freedom and complexity of design.^1−6^ In particular, for electroanalytical devices, it is becoming popular to employ multimaterial fusion filament fabrication (FFF) to construct electrochemical sensing devices.^7−11^ To that end, one nozzle prints a regular nonconductive thermoplastic, while the other nozzle is loaded with a conductive filament. Conductive filaments are composites made of a thermoplastic matrix, especially PLA, doped with carbon materials such as graphene (PLA-G),^12,13^ carbon nanotubes (PLA-CNT),^14,15^ or carbon black (PLA-CB).^8,16^

However, those conductive filaments are not intended to produce ready-to-use electrochemical sensors but rather conductive tracks for low-voltage circuitry^17,18^ or conductivity sensing.^14,15^ To use these filaments for chemical sensing applications, they need to be activated to remove thermoplastic from the surface and overexpose the conductive material. There are plenty of postprinting activation procedures described in the literature that provided satisfactory results, such as chemical,^19^ electrochemical,^20^ sanding,^21^ oxygen plasma,^22^ laser ablation,^23^ or thermal annealing^24^ and combinations of them. There is a very recent report that explored chemical pretreating of the filament before printing.^25^

Besides, FFF is a suitable technique for the fabrication of microfluidic devices because they can be constructed automatically in a single step employing a wide range of thermoplastic materials with different physicochemical properties. However, when closing the channel, most of the activation procedures listed before or any the surface modifications of the filament-based electrodes are restricted mainly to electrodeposition. FFF is a versatile technique that even permits the so-called print–pause–print (PPP). By taking advantage of the layer-by-layer construction, the printing process can be paused midprint and integrate external components (e.g., pumps, valves magnets) that provide new functionalities to the object.^26^ Still, pausing the microfabrication has not been employed to tailor the electrode surface to construct high-performing and customized electrochemical (bio)sensors. During the pause, filament-based electrodes can be modified in several ways before closing the channel and not being accessible anymore (e.g., drop casting, sanding, electrodeposition, or organic solvent PLA removal).

Herein, taking this technological opportunity, we have exploited the fabrication of hybrid electrochemical fluidic fused filament fabricated devices (h-eF^4^Ds) with analytical purposes. First, we technically demonstrated the integration of a high-performing electrochemical cell using graphite screen-printing ink for the working and counter electrodes and a Ag|AgCl ink for the reference electrode and their higher electrochemical performance in comparison with as-printed and activated PLA-CB electrodes. Screen printing has been underexplored in conjunction with FFF.^27,28^ It has been employed here as an alternative to the cumbersome and one-by-one activation of 3D-printed electrodes as the composition of the inks is very well optimized for electrochemical sensor construction. Then, to exemplify the possibilities of the PPP approach, we constructed a Prussian Blue (PB)-based glucose microfluidic biosensor.

## 2 Materials and Methods

### 2.1 Materials and Equipment

Ferrocene methanol, potassium ferrocyanide, potassium ferricyanide, dopamine hydrochloride, iron chloride (III), Nafion 5% solution, glucose, glucose oxidase, and sodium hydroxide were purchased from Sigma-Aldrich.

A nonconductive polyethylene terephthalate glycol (PETg) (SmartMaterials, Spain) filament and a carbon black-filled poly(lactic acid) filament (PLA-CB, Protopasta CDP11705, Protoplant, Canada) were used to construct the electrochemical microfluidic devices. A cleaning filament (SmartMaterials, Spain) was employed to eliminate the remaining PLA-CB filament from the nozzle when switching filaments. Carbon (C2180626D6, Gwent Group, United Kingdom) and Ag|AgCl (C2130809D5, Gwent Group, United Kingdom) inks were employed in stencil printing.

A Cartesian Prusa i3MK3 (Prusa, Czech Republic) was employed for printing the devices, and a Silhouette Cameo 3 (Silhouette America, United States) cutter plotter was employed to pattern the disposable vinyl masks for stencil printing. Electrochemical measurements were carried out in an EmStat4S (Palmsens, The Netherlands) controlled by PSTrace Software. A syringe pump (Pump 33, Harvard Apparatus) was employed to control the flow rate in the hydrodynamic experiments.

### 2.2 Device Design

The h-eF^4^Ds were designed using Fusion 360 (Student License, Autodesk, United States) like our previous work^16^ but with slight modifications. The dimensions of the grooves for stencil printing the electrodes width, length, and thickness were 2 × 2 × 0.4 mm (width × length × height) with a 5 mm pitch separation. On the other hand, microchannel width, height, and length were 1.5 mm, 0.4 mm, and 24 mm. The design was saved in STL format and sliced using PrusaSlicer (Prusa, Czech Republic) using the following settings: 0.2 mm layer height, 100% infill percentage, 1.1 extrusion multiplier, 230 °C nozzle temperature, 90 °C bed temperature, and 25 mm/s print speed.

### 2.3 PPP Fabrication of the h-eF^4^Ds

**Figure 1:**
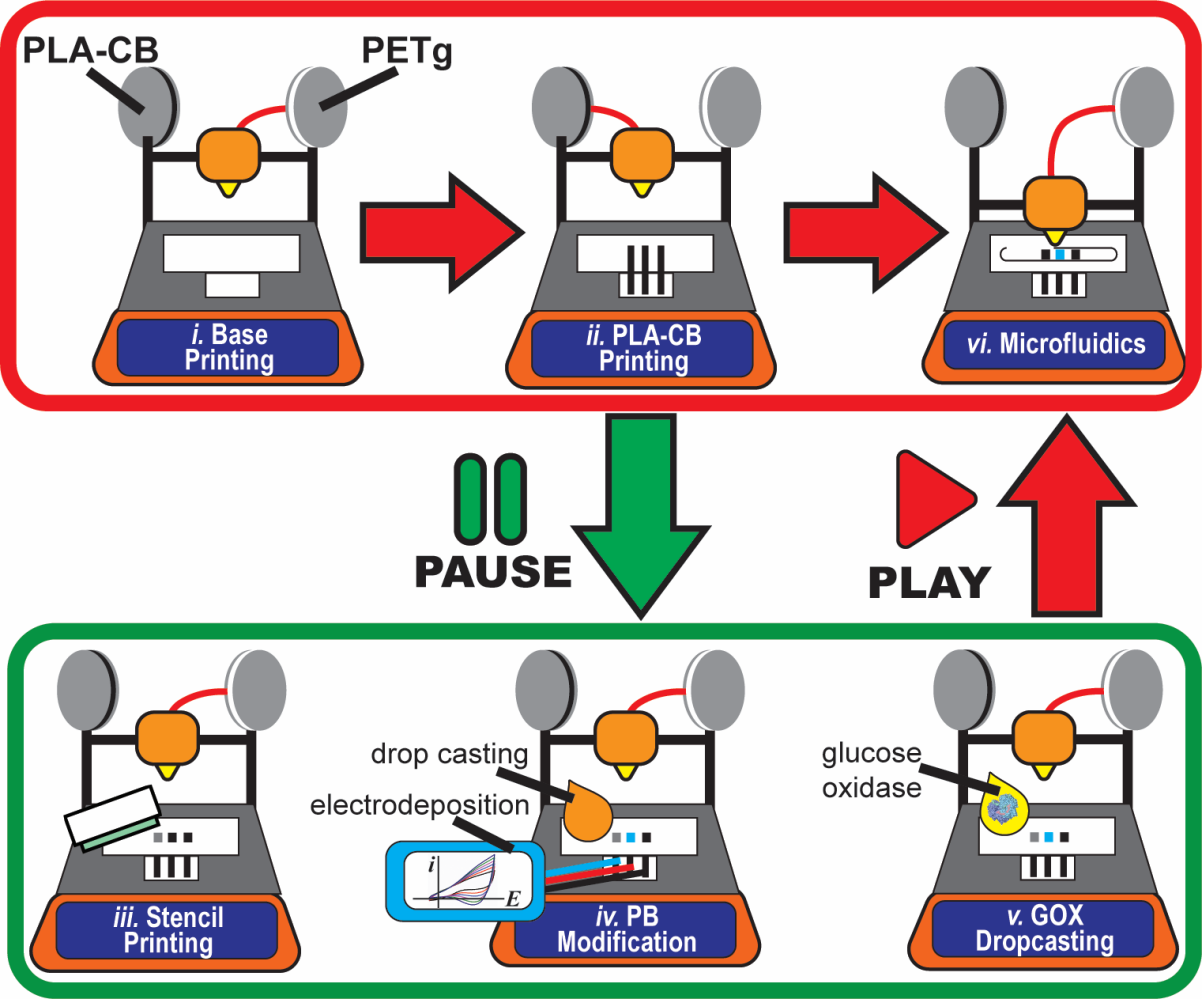
Overview of FFF of electrochemical microfluidic devices. The top panel (red) illustrates the fabrication of eF^4^Ds: base printing (i), PLA-CB printing (ii), and microfluidics (vi). The bottom panel (green) displays the print–pause–print operations: stencil printing (iii), nanomaterial modification (iv), and biofunctionalization (v) for the construction of electrochemical microfluidic devices.

The h-eF^4^Ds were prepared by combining FFF 3D printing and stencil printing steps. FFF was used for the microfluidics and structural elements employing a PETg filament and PLA-CB filament to pattern the electrodes. In the first stage, the lower cover of the device (PETg) and the conductive tracks (PLA-CB) were printed, switching the filament manually when employing a cold pull approach (Figure 1i). Next, on top of the current collectors, small square grooves were printed in PETg. This layer insulated the contacts and, at the same time, was used to pattern the electrodes in the following step. At this point, printing was paused for stencil printing. Vinyl masks matching the grooves were attached to the devices. Then, the carbon and Ag|AgCl inks were carefully stencil printed using a spatula. The ink was allowed to dry at room temperature overnight before modifying the electrodes further (see section 2.4). Finally, printing was resumed to print the microchannels, the sealing, and chip-to-world connectors.

### 2.4 Prussian Blue and Glucose Oxidase on-Bed Modification of the Working Electrodes

Carbon-based working electrodes were modified with PB by electrodeposition and drop casting. Briefly, for PB electrodeposition, a solution containing 2.5 mM Fe^3+^ and 2.5 mM [Fe(CN)_6_]^3–^ in 0.1 M KCl; 0.1 M HCl was cycled between +0.4 and +0.8 V for 10 cycles.^29^ PB electrodeposition was directly performed on the 3D printing bed employing a custom magnetic connector (see Supporting Information for more details). PB drop casting was performed by spotting a drop of 3 μL onto the working electrode containing 100 mM Fe^3+^ and 100 mM [Fe(CN)_6_]^3–^ in 0.1 M KCl, 0.1 M HCl. The electrode was then thoroughly rinsed in 0.1 M KCl, 0.1 M HCl.^30^ To improve the stability of the PB layer, 0.5% (v/v) Nafion was drop cast on the working electrode.

Glucose electrochemical biosensors were constructed upon PB-modified carbon electrodes. The method consisted of a two-step drop casting. First, a 3 μL drop of a 20 mg/mL glucose oxidase solution was cast on the working electrode and allowed to evaporate. Then, 0.5% (v/v) Nafion was drop casted to improve the stability of the sensor.

## 3 Results and Discussion

### 3.1 Dimensional Accuracy of the Fabrication Techniques

Figure 1 displays a scheme of the PPP fabrication of h-eF^4^Ds (for more details see Materials and Methods). The first step (Figure 1i) is to print the base layer of the device comprising nonconductive filament (PETg) to seal the bottom part of the devices and then PLA-CB-based conductive tracks (Figure 1ii). At this point, the fabrication can be either automatically continued to obtain eF^4^Ds based exclusively on PLA-CB electrodes (red panel)^16^ or strategically paused to make operations on the PLA-CB electrodes (green panel). This pause enabled us to perform stencil printing, drop casting, electrodeposition, and biofunctionalization before closing the channels. By using our PPP approach, PLA-CB electrodes were used as current collectors and were further modified with custom materials that are not available for printing. Graphite and Ag|AgCl conductive inks were stencil printed on top of PLA-CB filament to obtain ready-to-use as-printed sensors (Figure 1iii). After stencil printing there are several possibilities to continue the fabrication. The printing process could be directly resumed (Figure 1vi); hence, carbon-based h-eF^4^Ds were obtained, and the working electrode could be modified either by drop casting or by electrodeposition (Figure 1iv) with, for example, a nanomaterial such as Prussian Blue. The PB-based material could be further biofunctionalized (Figure 1v) with glucose oxidase to obtain electrochemical glucose biosensors. Regardless of the modification being carried out, microchannel printing is the last step because afterward there would be no physical access to the electrodes, making their modification more challenging.

The accuracy of the different microfabrication techniques employed (i.e., xurography, stencil printing, and FFF) was evaluated to design the devices accordingly. To that end, they were challenged to produce square grooves to test the smallest attainable resolution for the stencil-printed electrodes. Figure S1 shows the agreement between the CAD dimensions and the measured dimensions (MD) of the fabricated objects. Xurography (Figure S1A) and stencil printing (Figure S1B) exhibited high linear correlations (*R*^2^ = 0.998 and 0.999) in the 0.5–2 mm range with regression equations MD_xurography_ (μm) = (100 ± 20) + (0.94 ± 0.02) [CAD] (μm) and MD_stencil-printing_ (μm) = (150 ± 10) + (0.96 ± 0.01) [CAD] (μm) with errors expressed as the standard deviation, respectively (*n* = 9). However, in this case, FFF was the limiting fabrication technique (Figure S1C). FFF grooves smaller than 1 × 1 mm could not be fabricated in a reproducible manner using a 0.4 mm nozzle. In any case, FFF printing also presented good linearity (*R*^2^ = 0.992) with a regression line MD_FFF_ (μm) = (−380 ± 100) + (1.20 ± 0.06) [CAD] (μm) in the 1–2 mm range (*n* = 9). From these results, the stencil-printed electrodes were designed with the following dimensions 1.5 × 1.5 × 0.4 mm (width × length × height) employing the calibration plot lines. Figure S2 shows a picture of the PPP device.

### 3.2 Electrochemical Performance of Carbon-Based h-eF^4^Ds

Stencil printing inks require specific curing conditions to obtain their best electrochemical performance. Curing conditions often require high temperatures, which 3D printing materials cannot resist. Therefore, the optimal curing conditions were studied to obtain the best electrochemical behavior while preventing the 3D-printed model from thermal deformation (Figure S3). First, the printing bed was employed as a hot plate by heating the bed at 120 °C. However, this process did not provide good results with poor peak separation potential (Δ*E* = 475 ± 5 mV) regardless of the curing time. As heated on-bed thermal curing was proven not to be effective, the curing process was performed overnight directly on the printing bed without heating as an alternative. The devices displayed an acceptable peak separation potential (Δ*E* = 170 ± 20 mV) and peak current (6.9 ± 0.8 μA). We observed the same results if the printing was resumed right after the stencil-printing step and channels were closed while the curing process occurred. Nevertheless, this procedure was only employed for the fabrication of carbon-based electrodes without further modifications since the microchannel printing step enclosed the electrodes.

**Figure 2:**
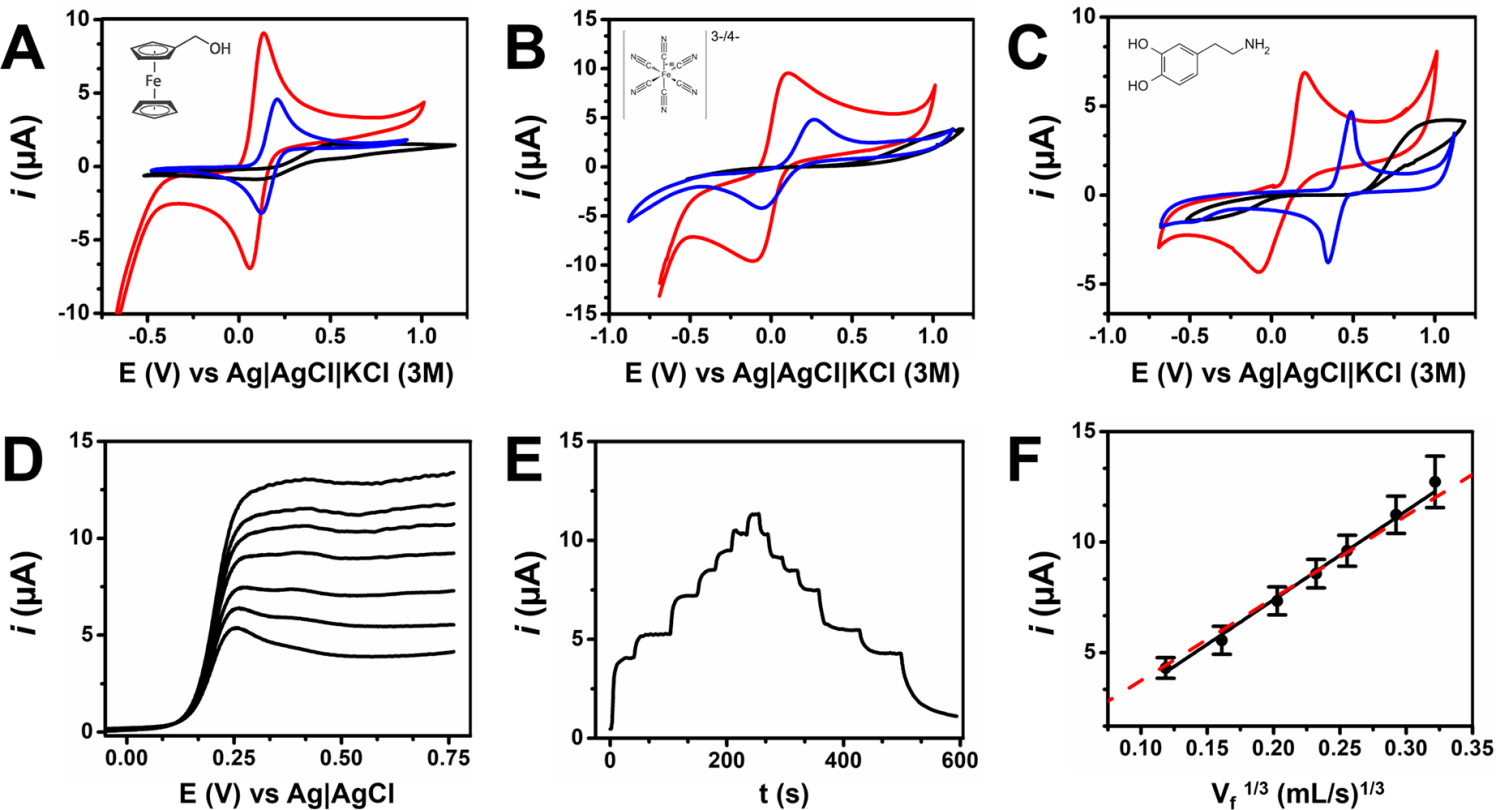
Electrochemical performance of stencil-printed carbon electrodes (red), electrochemically activated PLA-CB electrodes (blue), and as-printed nonactivated PLA-CB (black) under static conditions toward (A) 1 mM ferrocene methanol, (B) 1 mM ferro/ferricyanide, and (C) 1 mM dopamine. Conditions: (A and B) 0.1 mM PBS and (C) 0.1 M KCl/HCl. (D) LSV in the presence of 1 mM FcMeOH at increasing flow rates 100, 200, 500, 750, 1000, 1500, and 2000 μL/min. (E) Constant potential amperometry at those flow rates. Conditions: 0.4 V. (F) Linear relationship between limiting current and *v*_f_^1/3^ according to Levich-type equation. Points represent the experimental data, and the dashed line (red) represents the theoretical values. Error bars correspond to *n* = 3 devices.

The electrochemical response of the h-eF^4^Ds toward three redox-active molecules (i.e., ferrocene methanol, ferrocyanide, and dopamine) was compared with fully 3D as-printed and electrochemically activated eF^4^Ds in which electrodes were composed exclusively of PLA-CB (Figure 2). The peak separation potential and peak current were improved in h-eF^4^Ds compared with 3D as printed. Activated 3D-printed electrodes showed an improved performance to 3D as-printed eF^4^Ds; however, a smaller current and higher peak separation potential were found for ferrocene methanol and ferrocyanide compared to h-eF^4^Ds. This is especially noticeable for ferrocene methanol, an outer-sphere compound, where the surface chemistry has less influence in the electrochemical signal. In contrast, for a surface-sensitive compound like in the case of dopamine, the peak separation potential was better for the activated 3D-printed electrodes than the h-eF^4^Ds (150 vs 250 mV), whereas smaller peak currents were registered. An explanation for this finding is that during the electrochemical activation of the PLA-CB electrodes oxygenated groups were incorporated onto the working electrode surface that electrocatalyzed the oxidation/reduction of dopamine.^31^ However, the eF4Ds showed a general improved performance for the selected widely used electrochemical probes.

It is important to highlight that the electrochemical activation procedure was performed one by one as it involved a cyclic voltammetry treatment in 0.5 M NaOH,^16^ which is a labor-intensive approach. Stencil printing of multiple devices can be performed at the same time, and as mentioned above, the curing of the ink can take place while microchannels are printed. This showcased that stencil printing is a suitable method to obtain numerous electrochemical microfluidic devices with great electrochemical performance in shorter cycle times.

Miniaturization of the reference electrode is always a challenge for microfluidic devices. As there are still no commercial filaments able to act as a reliable reference electrode, PLA-CB reference electrodes have been normally employed in 3D-printed microfluidic electroanalytical devices. Ag|AgCl pseudoreference electrodes have been only used by inserting a Ag|AgCl wire in the outlet tube^10^ or by electrodeposition.^32^ Stencil printing, which simplified the introduction of Ag|AgCl pseudoreference electrodes, has been only used in a batch approach in which the ink can be deposited after printing all of the parts^28^ and in a potentiometric microfluidic platform in which the ink was deposited midprinting like in this work.^27^Figure S4 displays the cyclic voltammetry results employing different materials as reference electrodes, carbon, Ag|AgCl inks, and an external Ag|AgCl|KCl (3M) commercial reference electrode. Hereafter, h-eF^4^Ds were fabricated employing the Ag|AgCl ink for the reference electrode because its potential is more stable than that of a carbon reference electrode, and it is miniaturizable in contrast to the commercial Ag|AgCl|KCl (3M).

The performance of the carbon-based h-eF^4^Ds was also studied under hydrodynamic conditions employing FcMeOH. The PPP approach produced leak-free monolithic devices even when the printing was paused overnight to allow the curing of the ink. Figure 2D displays the development of an increasing limiting current with the increasing flow rate due to the improved mass transfer to the electrode surface under flowing conditions. Then, the relationship between the limiting current and the flow rate was studied in constant potential amperometry (Figure 2E). The limiting current was stable over time for each flow rate and increased immediately as the flow rate changed. The limiting current registered was plotted against the cubic root of the flow rate, and the values fitted the predicted values from the Levich-like model for microband electrodes in microchannels (Figure 2F): *i*_Levich_ = 0.925*nFC*_0_(*Dwx*/*h*)^2/3^*v*_F_^1/3^, where *n* is the number of electrons, *F* is Faraday’s constant, *C*_0_ is the bulk concentration of the species, *D* is the diffusion constant, *w* is the microchannel width, *x* is the width of the electrode, *h* is one-half of the height of the microchannel, and *v*_f_ is the average velocity. Besides, intra- and interelectrode repeatability was studied employing FcMeOH in flow injection analysis (FIA) for *n* = 9 devices. The excellent CV for intra (<6%) and interelectrode (7%) repeatability confirmed the suitability of this fabrication approach.

### 3.3 Electrochemical Performance of Modified Carbon-Based h-eF4Ds: Prussian Blue and Glucose Oxidase

Carbon-based working electrodes have been extensively modified with PB for the construction of enzyme-mediated biosensors due to their great selectivity toward H_2_O_2_ reduction. It can be prepared in several ways, such as drop casting or electrodeposition, which is highly suitable for demonstrating the versatility of the proposed fabrication technique. PB electrodeposition was the first approach employed for modifying working electrodes. As electrodes must be modified on the printing bed and taking advantage of the magnetism of the 3D printing bed, a customized magnetic holder with three pogo pin connectors was built (Figure S5). Unfortunately, during the electrodeposition, it was observed that PB was also formed at the Ag|AgCl reference electrode. Therefore, an external Ag|AgCl (KCl 3 M) reference electrode was employed during the electrodeposition to avoid modifying the sensing reference electrode integrated into the device. PB electrodeposition was also tested on as-printed PLA-CB. However, an almost negligible amount of PB was electrodeposited (data not shown). The main advantage of electrodeposition over drop casting is that it can be performed even with a closed channel. However, in that case, the working electrode cannot be selectively modified via drop casting with biorecognition elements. In contrast to electrodeposition, a full batch of electrodes was modified simultaneously via drop casting, thus accelerating the development of PB h-eF^4^Ds.

**Figure 3:**
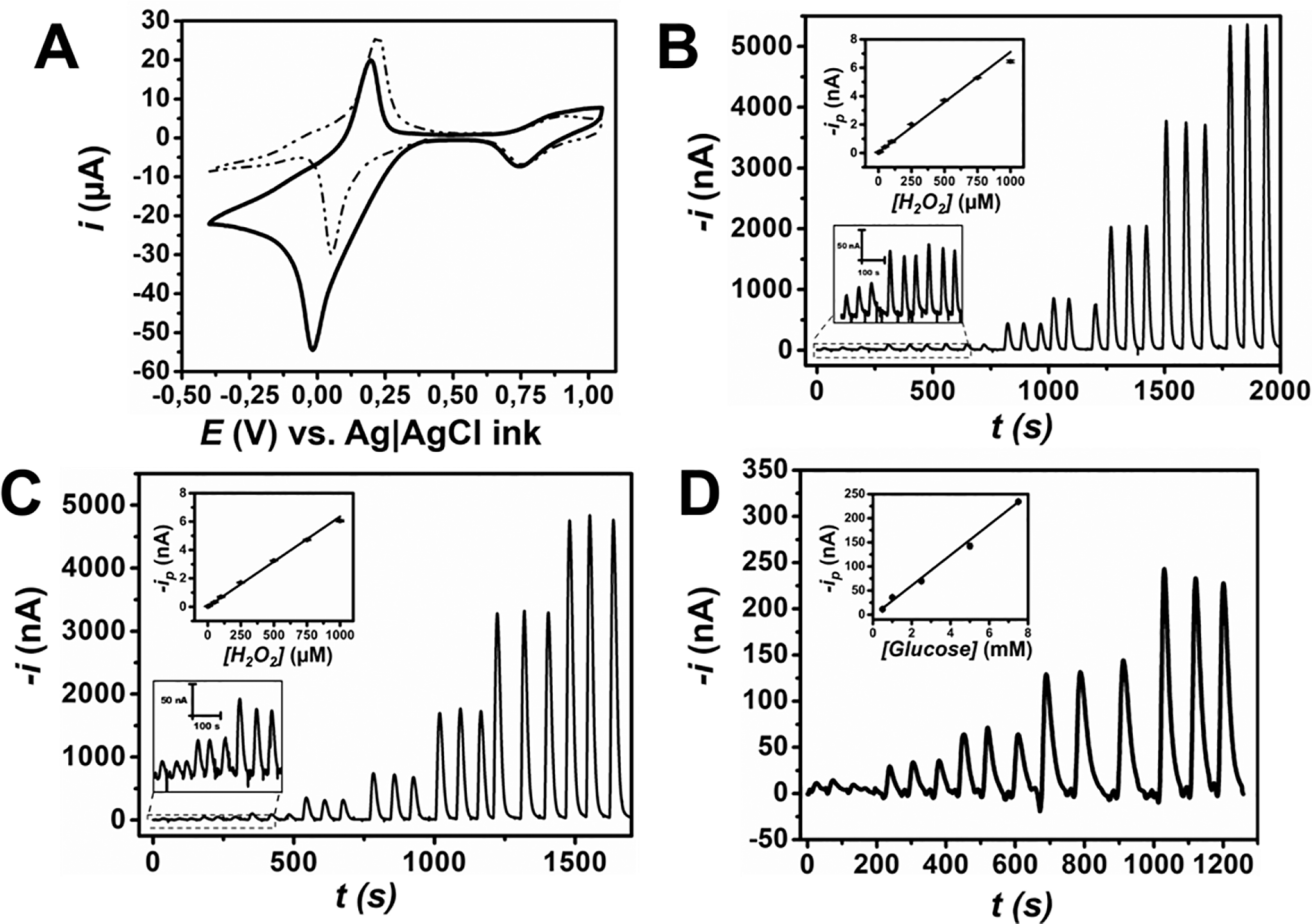
Electrochemical performance of the modified carbon electrodes. (A) Cyclic voltammogram obtained at 10 mV s^–1^ in 0.1 M HCl/KCl in the absence (dashed) and presence of 5 mM H_2_O_2_ (solid) at drop-casting PB-modified electrodes. Calibration of H_2_O_2_ in 0.1 M PBS at (B) electrodeposited PB-modified h-eF^4^Ds under FIA and (C) drop-cast PB-modified h-eF^4^Ds. The bottom insets display the lower calibration points, and the upper ones display the calibration plots. (D) Calibration of glucose in glucose oxidase/PB-modified eF^4^Dfs under FIA. Conditions: 1000 μL min^–1^, *E* = 0 V. Error bars correspond to *n* = 3 devices.

The presence of PB on the surface of the working electrode and its response toward H_2_O_2_ reduction was first confirmed using CV (Figure 3A). Then, the hydrodynamic behavior of PB-modified h-eF^4^Ds by electrodeposition and drop casting was characterized for the determination of H_2_O_2_ by flow injection analysis (FIA). First, the stability of PB under these conditions was evaluated. To that end, cyclic voltammograms were registered before and after 10 injections of H_2_O_2_ under FIA to determine the quantity of PB remaining on the electrode surface. Figure S6A shows that there is a dramatic decrease in PB content after FIA, even in acidic conditions at which PB remains more stable. Nonetheless, this reduction in PB content was not observed in PB-modified devices with the Nafion membrane. In Figure S6B, it can be observed that the content of PB remained stable even after FIA. Hence, Nafion coating is essential for PB-modified h-eF^4^Ds under flowing conditions. Even if PB could be prepared by electrodeposition in closed devices, the Nafion protective membrane must be drop cast. Thus, the PPP approach is totally necessary to construct monolithic 3D-printed electrochemical microfluidic devices with improved long-term behavior.

PB-modified devices via electrodeposition presented a regression equation *i*_p_ (nA) = (50 ± 30) + (7.1 ± 0.1) [H_2_O_2_] with excellent linearity (*R*^2^ = 0.998) (Figure 3B). Similarly, PB-modified h-eF^4^Ds by drop casting also presented great linearity (*R*^2^ = 0.999) and a regression equation *i*_p_ (nA) = (11 ± 2) + (6.42 ± 0.07) [H_2_O_2_] (Figure 3C). Given the results, drop casting was selected as the method to modify carbon electrodes with PB hereafter, as multiple devices can be modified simultaneously, yielding identical electrochemical performance to the device-by-device PB electrodeposition.

Finally, PB-modified carbon electrodes were biofunctionalized with glucose oxidase to construct glucose biosensors. Glucose oxidase was drop cast on top of the PB layer, and then, it was coated with Nafion to trap the enzyme and improve the stability of the biosensor under hydrodynamic conditions. Figure 3D displays the calibration for glucose determination in 0.1 M PBS with great linearity (*R*^2^ = 0.998) in the 0.5–7.5 mM range and regression equation *i* (nA) = (0 ± 4) + (31 ± 1) [Glucose] (mM), with errors expressed as the intercept (*S*_a_) and slope (*S*_b_) standard deviations. The limit of detection and quantification were 0.1 and 0.5 mM, which were calculated as 3 and 10 s/m, respectively, where *s* is the standard deviation of the lowest concentration of the calibration and *m* is the slope of the calibration line.

## 4 Conclusion

In comparison to conventional microfabrication approaches, in which alignment and bonding steps are required, FFF can produce monolithic electrochemical microfluidic devices in a single step. This is an advantage for microfluidics; however, physical access to the electrodes is lost during the printing process. Hence, they cannot be easily modified and/or (bio)functionalized, limiting dramatically its analytical potential. Pausing the printing process in an elegant and creative manner allowed us to perform stencil printing, electrodeposition, and drop casting on the electrodes before closing the microchannels. Interestingly, we demonstrated that the subsequent 3D printing of the channels deteriorated neither the performance of the conductive inks nor the (bio)functionalization of the electrode surface.

Although the PPP approach described here could be seen as a step back in the automatization of the technique, it opens new creative paths in the functional use of these devices with a realistic potential in (bio)sensing applications. Furthermore, we believe that the number and type of operations could be even larger (e.g., polishing, laser ablation, or direct ink writing). We also believe that this technology will facilitate the implementation of all of the accumulated knowledge in electrochemical (bio)sensor design and construction and integrate it into high-throughput customized 3D-printed devices.

## Supporting Information Available

Dimensional accuracy studies, optimization of the on-bed curing conditions, potential shift with different reference materials, experimental details for the on-bed electrodeposition, and effect of Nafion membrane under hydrodynamic conditions (PDF)
