# Supplementary material for: Print–Pause–Print Fabrication of Tailored Electrochemical Microfluidic Devices
Source: Anal Chem. 2023 Dec 14;95(51):18679–84. doi: 10.1021/acs.analchem.3c03364 (PMC10753525; doi:10.1021/acs.analchem.3c03364)
Supplement: Supplementary file 1 — ac3c03364_si_001.pdf [file ac3c03364_si_001.pdf]

Print-pause-print fabrication of tailored electrochemical microfluidic devices

Juan F. Hernández-Rodríguez[a], Daniel Rojas*[a] and Alberto Escarpa*[a, b]

[a] *Department of Analytical Chemistry, Physical Chemistry and Chemical Engineering, University of Alcalá, Alcalá de Henares, 28805, Madrid, Spain.*

[b] *Chemical Research Institute "Andres M. Del Rio", University of Alcalá, Alcalá de Henares, 28805, Madrid, Spain*

Contents

1 Dimensional accuracy of the fabrication techniques 2
2 Photographs from the Print-Pause-Print devices 3
3 Effect of on-bed curing conditions on the electrochemical performance 4
4 Potential shift with respect to the reference electrode 5
5 Intra- and inter-electrode repeatability 6
6 Electrodeposition on the printing bed 7
7 Effect of Nafion membrane under hydrodynamic conditions 8

1 Dimensional accuracy of the fabrication techniques

Xurography, stencil printing and fused filament fabrication were employed in this work to fabricate the square grooves that defined the stencil printed electrodes area on top of the PLA-CB electrodes. To optimize the minimum attainable resolution for the electrodes, grooves (n=10) with dimensions ranging from 0.5 mm to 2 mm were fabricated and their dimensions were measured employing ImageJ.

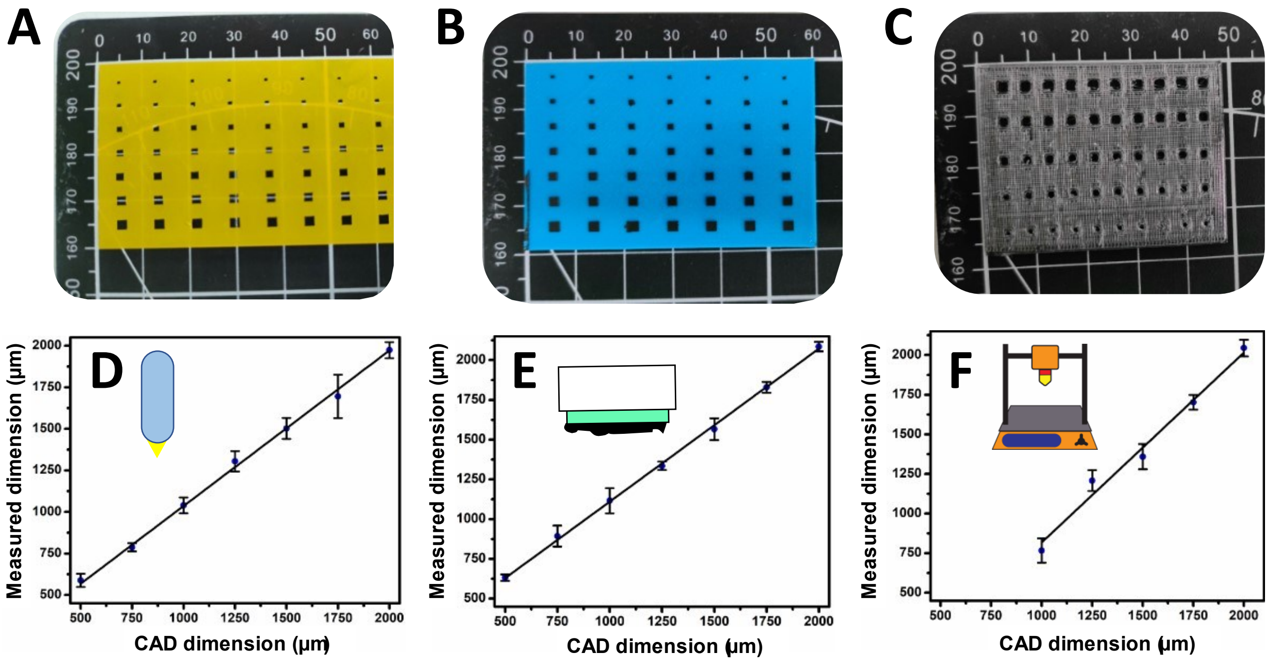

Figure S1. Optimization of the attainable electrode resolution. Photographs from the grooves were produced using xurography (A), stencil printing (B), and fused filament fabrication (C). Correlation of the measured dimensions in A-C to the CAD design for xurography (C), stencil printing (D), and fused filament fabrication (F). Error bars correspond to 10 square grooves.

2 Photographs from the Print-Pause-Print devices

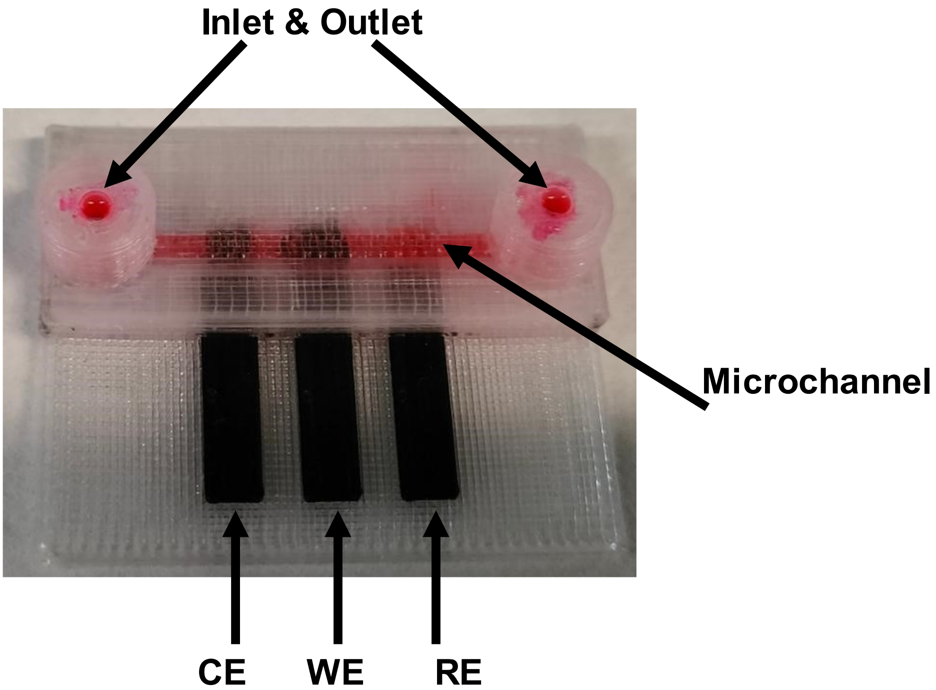

Figure S2. Hybrid electrochemical fluidic fused filament fabricated device filled with rhodamine.

3 Effect of on-bed curing conditions on the electrochemical performance

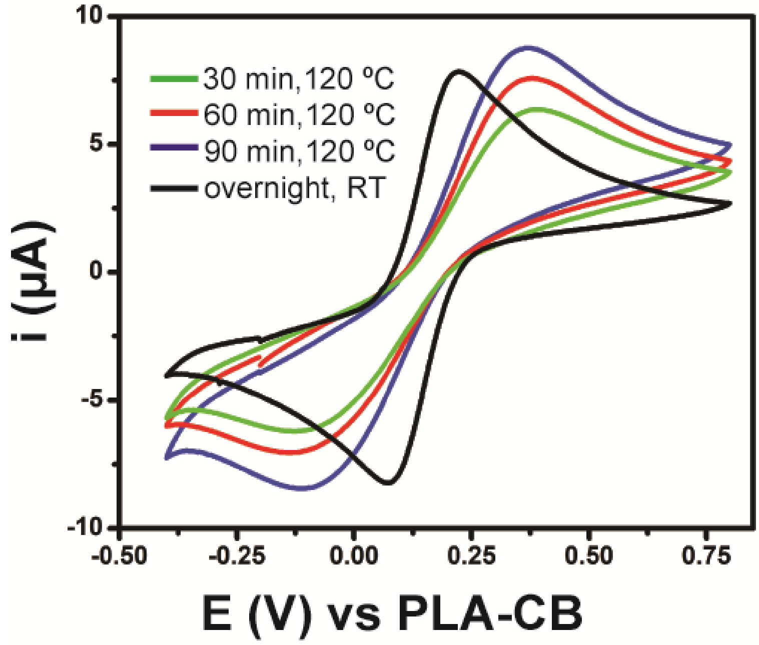

Figure S3. Cyclic voltammetries in 1 mM $[Fe(CN)_6]^{3-/4-}$ for eF^{4}Ds cured on the printing bed at 120ºC for 30 min (green), 60 min (red), 90 min (blue) and overnight at room temperature (black).

4 Potential shift with respect to the reference electrode

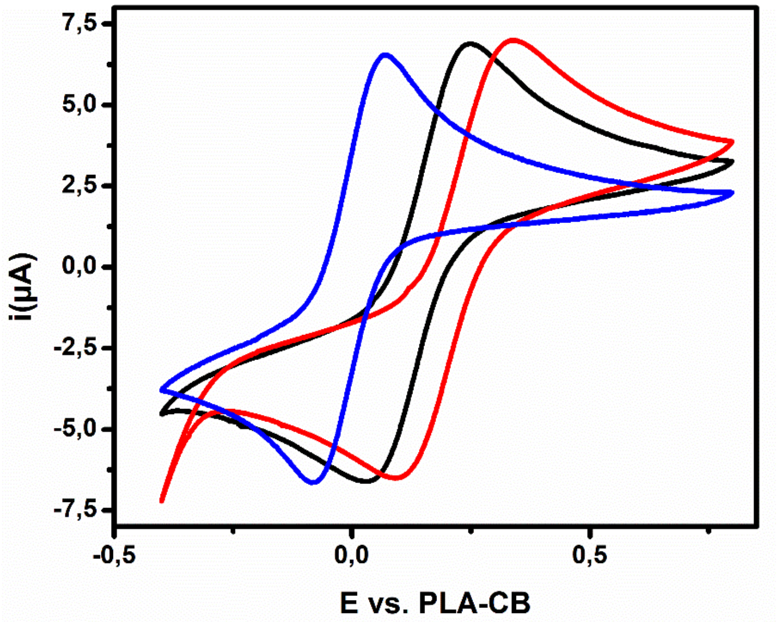

Figure S4. Potential shifting with the different reference electrodes employed: carbon (blue), Ag/AgCl screen printing ink (black) and external Ag|AgCl|KCl (3M) (red) in the presence of 1 mM $[Fe(CN)_6]^{3-/4-}$. Buffer: 0.1M PBS.

5 Intra- and inter-electrode repeatability

Intra- and inter-electrode reproducibility was studied by means of flow injection analysis employing n = 9 devices. To that end, six plugs of 1mM ferrocene methanol were injected per device and the oxidation current was registered at +0.30 V. The coefficient of variation of the intra-electrode repeatability was in all cases below 6% while the inter-electrode reproducibility was 7%.

Table S1. Intra- an interelectrode repeatability study (n=9)

Device	Peak current (µA)						Mean (µA)	Standard deviation (µA)	CV (%)
**1**	4.23	4.02	4.54	4.31	4.19	4.86	4.4	0.3	6
**2**	4.54	4.87	4.75	4.88	5.03	4.71	4.8	0.2	3
**3**	4.12	4.54	4.59	4.42	4.43	4.49	4.4	0.2	3
**4**	4.85	5.01	4.91	4.79	4.59	4.23	4.7	0.3	5
**5**	4.73	4.77	5.06	4.89	4.78	4.75	4.8	0.1	2
**6**	5.05	5.66	5.00	4.62	5.33	5.09	5.1	0.3	6
**7**	4.60	4.88	4.65	5.08	5.11	5.03	4.9	0.2	4
**8**	4.42	4.81	4.76	4.77	4.95	4.84	4.8	0.2	4
**9**	4.55	4.37	4.49	4.53	4.39	4.44	4.5	0.1	2
**Inter-electrode repeatability**							**4.7**	**0.3**	**7**

6 Electrodeposition on the printing bed

A customized magnetic connector was designed and fabricated to perform electrochemical methods on the 3D printing bed. For that purpose, a rectangular FFF PETg piece was printed with three holes (Ø 1 mm) matching the pitch of the electrodes in the device. Gold pogo pin connectors where inserted on each hole to serve as interface between the potentiostat wires and the eF4Ds. At each end circular neodymium magnets (Ø 5 mm, 1 mm thickness) magnets were glued to the PETg holder and held it in place on the bed during the electrochemical methods taking advantage of the magnetic nature of the printing bed. Pogo pins were interfaced with Dupont cables with the potentiostat connectors.

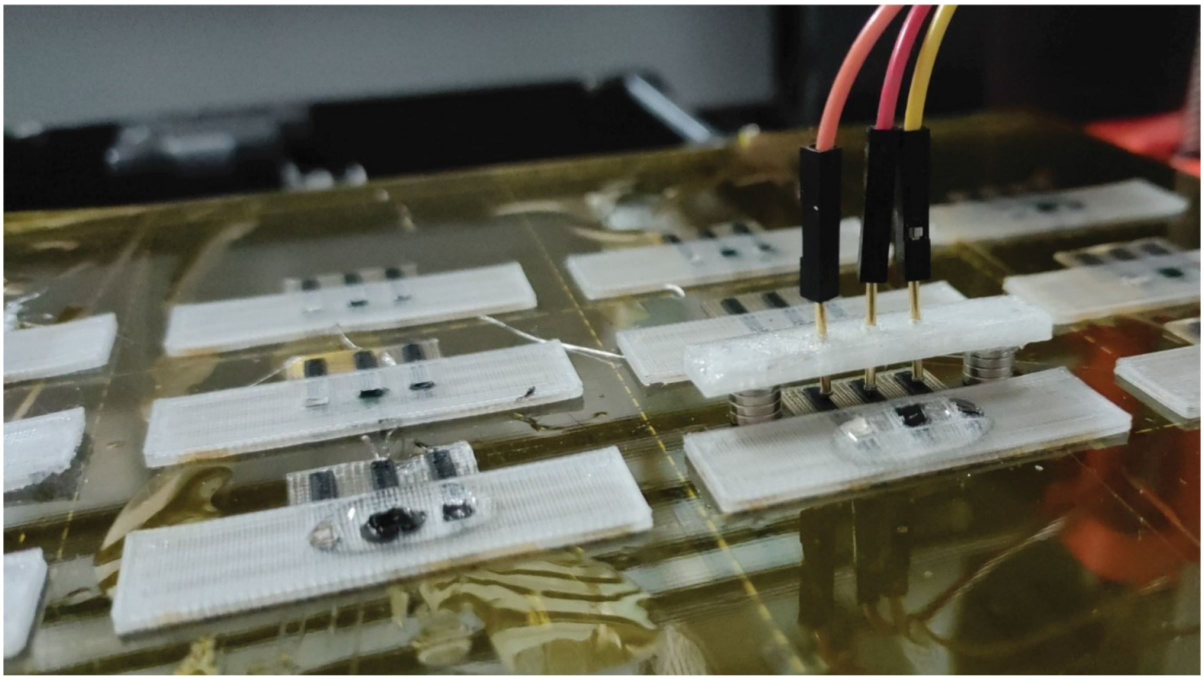

Figure S5. Electric connector for on-printing bed electrodeposition.

7 Effect of Nafion membrane under hydrodynamic conditions

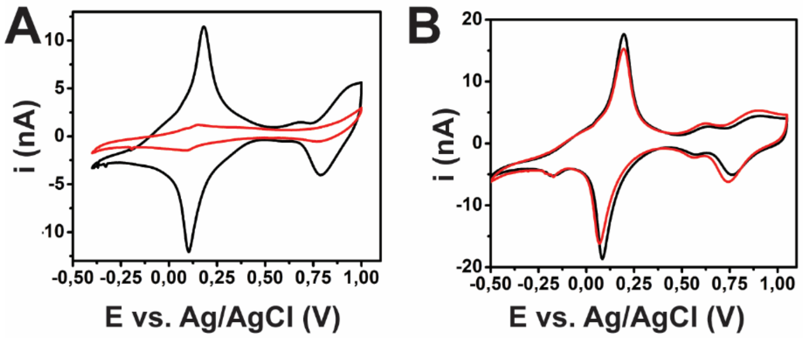

Figure S6. Effect of the Nafion layer under hydrodynamic conditions. (A) Cyclic voltammetry of PB modified EMDs without Nafion layer before (black) and after (red) flow injection analysis. (B) Cyclic voltammetry of PB modified eF^{4}Ds with Nafion layer before (black) and after (red) flow injection analysis. Buffer: 0.5 M HCl/KCl.
